# Supplementary material for: Conditional chemoconnectomics (cCCTomics) as a strategy for efficient and conditional targeting of chemical transmission
Source: eLife. 2024 Apr 30;12:RP91927. doi: 10.7554/eLife.91927 (PMC11060718; doi:10.7554/eLife.91927)
Supplement: Figure 3—source data 3. [file elife-91927-fig3-data3.zip › Figure 3 source data3 part 1.zip!/Figure 3-Source data 3-part1/1081-1-CRISPResso_Report_8zosk6yj.zip!/CRISPResso_on_1081_2/1c.Alignment_barplot.pdf]

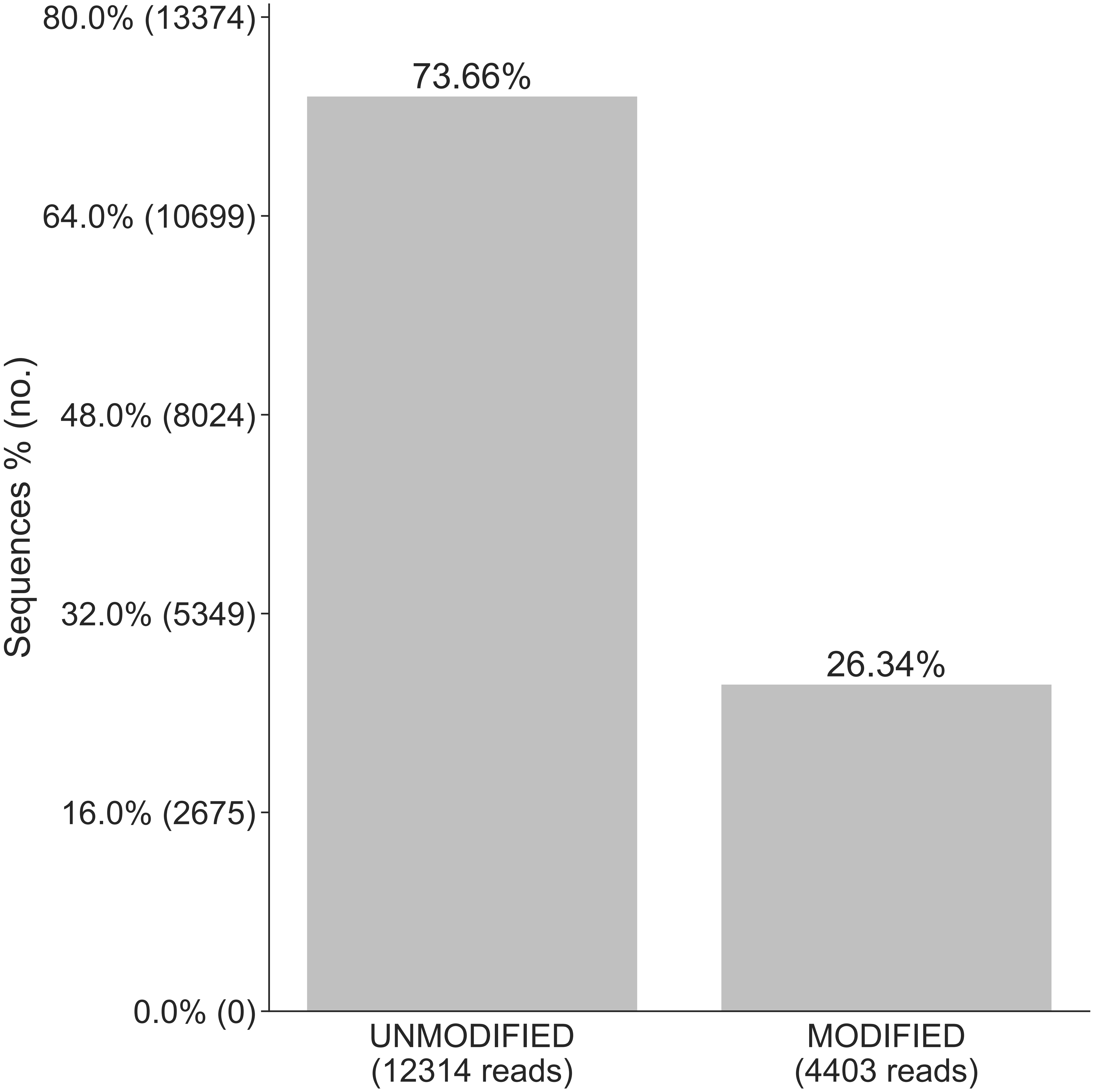

Sequences % (no.)
80.0% (13374)
64.0% (10699)
48.0% (8024)
32.0% (5349)
16.0% (2675)
0.0% (0)
73.66%
26.34%
UNMODIFIED
(12314 reads)
MODIFIED
(4403 reads)
